# A novel class of copper(II)- and zinc(II)-bound non-steroidal anti-inflammatory drugs that inhibits acute inflammation in vivo

**DOI:** 10.1186/s13578-016-0076-8

**Published:** 2016-02-06

**Authors:** Rajesh Puranik, Shisan Bao, Antonio M. Bonin, Ravinder Kaur, Jane E. Weder, Llewellyn Casbolt, Trevor W. Hambley, Peter A. Lay, Philip J. Barter, Kerry-Anne Rye

**Affiliations:** The Heart Research Institute, 7, Eliza St., Sydney, NSW 2042 Australia; The School of Chemistry, The University of Sydney, Sydney, NSW 2006 Australia; Casbolt & Associates Pty Ltd, Sydney, Australia; Lipid Research Group, Centre for Vascular Research, The University of New South Wales, Sydney, NSW 2052 Australia; Discipline of Pathology, D17, The School of Medical Sciences and Bosch Institute, The University of Sydney, Sydney, NSW 2006 Australia; Department of Cardiology, Royal Prince Alfred Hospital, Missenden Rd, Camperdown, NSW 2050 Australia

**Keywords:** NSAIDs, Indomethacin, Copper(II) indomethacin, Copper(II) ACM, Zinc(II) ACM

## Abstract

**Background:**

The ability of Zn(II) and Cu(II) metal complexes of non-steroidal anti-inflammatory drugs (NSAIDs) to inhibit acute arterial inflammation in vivo has been studied.

**Results:**

When acute vascular inflammation was induced in normocholesterolemic New Zealand White rabbits by inserting a non-occlusive silastic collar around the common carotid artery, a single oral dose of Cu(II)-indomethacin (Cu(II)Indo, 3 mg/kg) administered by laparotomy achieved a 67 % (8.2 ± 1.7 vs. 2.7 ± 0.4 image units, *p* < 0.05) reduction in endothelial expression of vascular cell adhesion molecule-1 (VCAM-1) but did not inhibit endothelial intercellular adhesion molecule (ICAM-1) expression significantly. Treatment with Cu(II)-acemetacin (Cu(II)ACM, 3 mg/kg) led to a profound 88 % (8.2 ± 1.7 vs. 1.0 ± 0.5 image units, *p* < 0.01) reduction in endothelial VCAM-1 expression but did not inhibit ICAM-1 expression, while treatment with Zn(II)-acemetacin (Zn(II)ACM, 3 mg/kg) led to an 84 % (19.3 ± 1.0 vs. 3.1 ± 1.2 image units, *p* < 0.01) reduction in endothelial ICAM-1 expression and did not inhibit VCAM-1 expression. No adverse gastric, hepatic or renal effects were observed in treated animals.

**Conclusion:**

These findings provide the “proof of concept” that this novel class of drug, where there is complexation of NSAIDs with metal ions, has substantial anti-inflammatory effects in an animal model of acute vascular inflammation with the possibility of low rates of adverse effects.

## Background

Non-steroidal anti-inflammatory drugs (NSAIDs) are amongst the most commonly prescribed drugs in the community, specifically for the treatment of a wide variety of inflammatory conditions [1]. However, patients receiving non-selective NSAIDs often experience abdominal discomfort, while some develop serious gastro-intestinal (GI) complications such as ulceration, bleeding, perforation or obstruction [2].

It was, therefore postulated that with the advent of selective cyclooxygenase-2 (COX-2) inhibitors, the number of NSAID-related gastric complications would be reduced [3]. However, current evidence demonstrates that selective COX2 inhibitors (‘coxibs’) have important cardiovascular side-effects that include increased risk for myocardial infarction, stroke, heart failure and hypertension [4]. Due to concerns regarding CV risks, the American Heart Association recommends that treatment with coxibs should be limited to patients in whom no appropriate alternatives can be found, and then only in the lowest dose for the shortest duration necessary [5]. A subsequent joint study with the American College of Cardiology Foundation and the American College of Gastroenterology recommended concomitant therapy with proton pump inhibitors such as esomeprazole, to offset the GI damage from NSAID use [6]. According to the Pharmaceutical Benefits Scheme, esomeprazole use represents the fourth highest cost to Australia in terms of national health expenditure.

An alternative approach, used in veterinary practice [7], is the complexation of NSAIDs with metal ions, which results in enhanced gastric protection [7] compared with the parent NSAID. Another potential strategy for developing NSAIDs with attenuated GI side effects is to use acemetacin (ACMH), a glycolic ester of indomethacin (IndoH) with inherent anti-inflammatory activity and better gastric tolerability compared with IndoH [8]. In addition, ACMH has a reportedly safer pharmacokinetic profile in the elderly [8]. Therefore, it is an ideal candidate for complexation with biologically relevant metals such as copper (Cu) and zinc (Zn) [9].

Given the pressing need for alternative safer NSAID-type drugs that could be applied to a large range of medical indications, we have investigated the impact of IndoH and copper bound Indo (Cu(II)Indo) with that of copper bound (Cu(II)ACM), and zinc bound ACM (Zn(II)ACM) on acute arterial inflammation in vivo in the New Zealand White (NZW) rabbit.

## Results and discussion

The mean plasma concentrations of total cholesterol, HDL cholesterol and apoA-I in the rabbits were 0.84 ± 0.1, 0.66 ± 0.05 mM/l, and 0.76 ± 0.07 mg/ml, respectively. At the time of sacrifice, there was no measurable effect on the concentration of any of these parameters with any of the treatments that were administered.

### Effect of treatment on endothelial VCAM-1 expression (Fig. 1a–g)

**Fig. 1:**
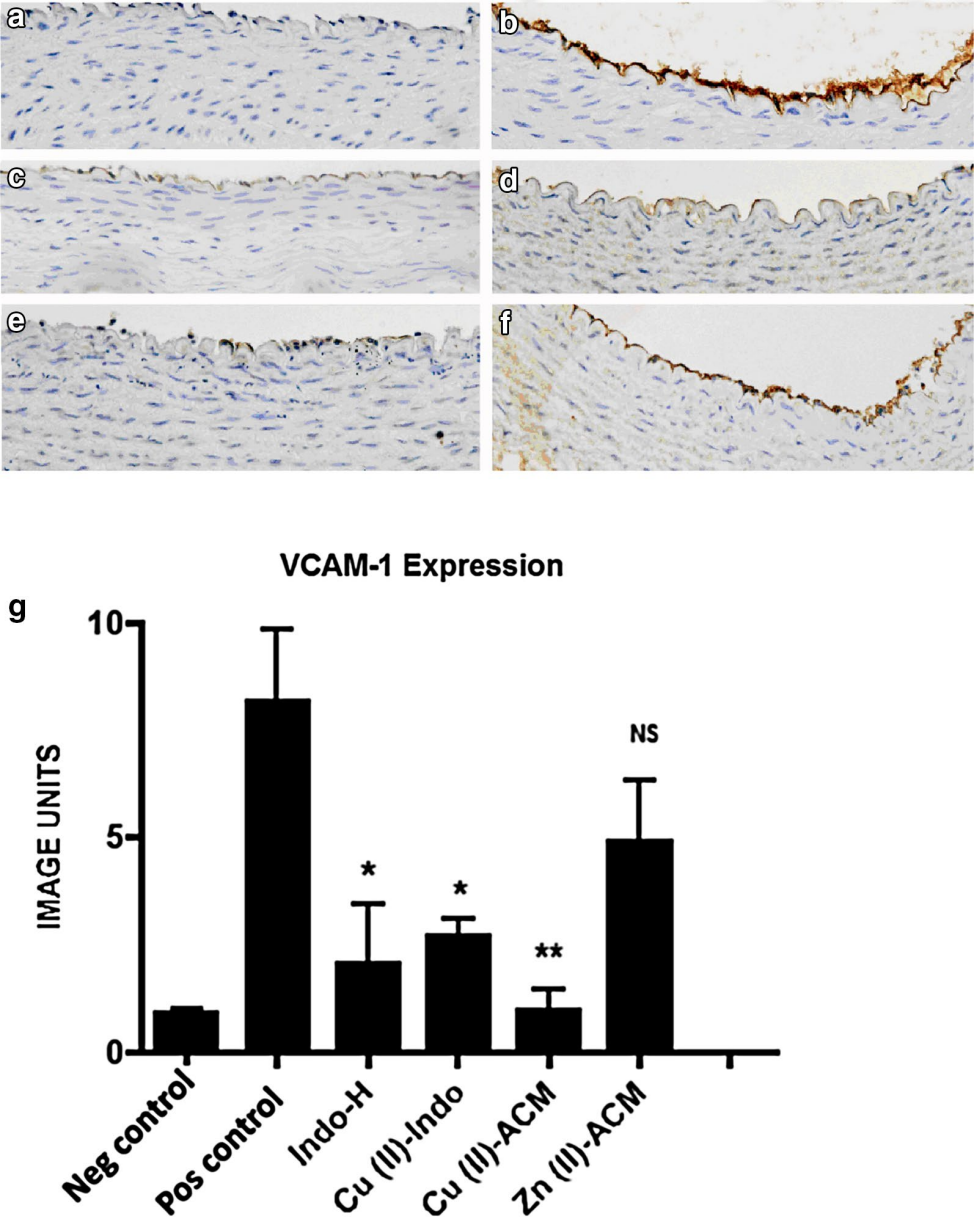
Effect of treatment on endothelial expression of VCAM-1. Normocholesterolemic NZW rabbits were treated with either saline, IndoH (3 mg/kg), Cu(II)Indo (3 mg/kg), Cu(II)ACM (3 mg/kg) or Zn(II)ACM (3 mg/kg) (*n* = 4 per group) at the time of peri-arterial collar insertion. The animals were sacrificed 24 h after collar insertion. Representative sections of carotid arteries stained for VCAM-1 expression (×40 magnification) are shown. **a** a representative control, non-collared artery from a saline-treated animal, **b** a collared artery section from a saline-treated animal, **c** a section of a collared artery where the animal is treated with IndoH, **d** a section of a collared artery from an animal treated with Cu(II)Indo, **e** a collared artery section from an animal treated with Cu(II)ACM and **f** a section of a collared artery from an animal is treated with Zn(II)ACM. In **g** the results are expressed graphically as mean ± SEM (*p < 0.05; **p < 0.01)

There was marked up regulation in endothelial expression of VCAM-1 24 h post-carotid collar insertion (Fig. 1a, b, *p* < 0.01). Treatment with IndoH (3 mg/kg) led to a 75 % decrease in endothelial VCAM-1 expression (8.2 ± 1.7 vs. 2.1 ± 1.4 image units) (*p* < 0.05) (Fig. 1c). Treatment with Cu(II)Indo (3 mg/kg) led to a 67 % (8.2 ± 1.7 vs. 2.7 ± 0.4 image units) (*p* < 0.05) reduction in endothelial VCAM-1 expression (Fig. 1d). Treatment with Cu(II)ACM (3 mg/kg) resulted in an 88 % (8.2 ± 1.7 vs. 1.0 ± 0.5 image units) (*p* < 0.01) reduction of endothelial VCAM-1 expression (Fig. 1e). The 39 % (8.2 ± 1.7 vs. 5.0 ± 1.4 image units, *p* = 0.19) reduction in VCAM-1 expression, which resulted from treatment with Zn(II)ACM (3 mg/kg), did not achieve statistical significance (Fig. 1f).

### Effect of treatment on endothelial ICAM-1 expression (Fig. 2a–g)

**Fig. 2:**
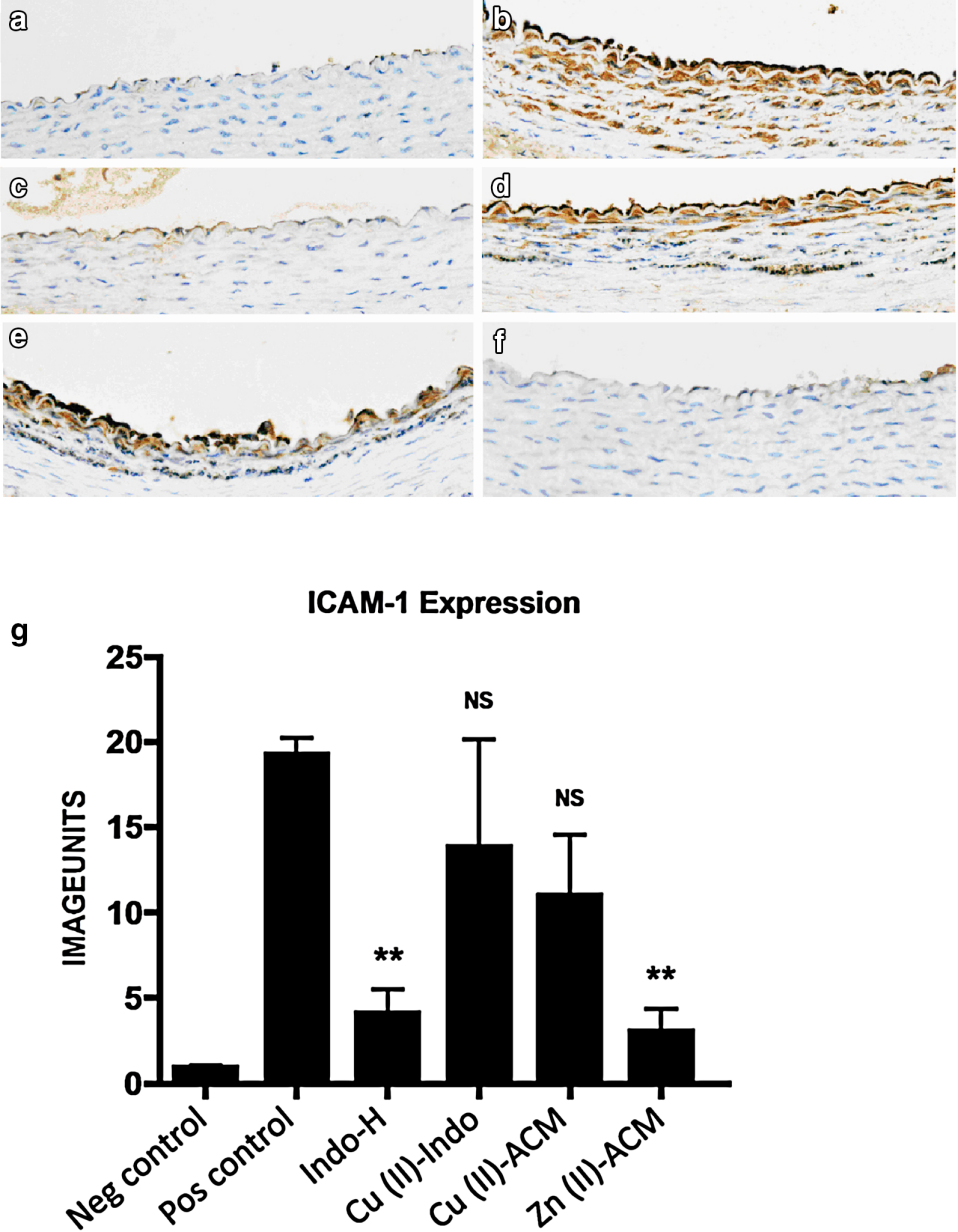
Effect of treatment on endothelial expression of ICAM-1. A non-occlusive collar was inserted around the left common carotid artery of normolipidemic NZW rabbits, as described in the legend to Fig. 1. Representative sections of carotid arteries stained for ICAM-1 expression (×40 magnification) are shown in the panels. **a** a representative section from a control, non-collared artery from a saline-treated animal, **b** a collared artery section from a saline-treated animal, **c** a section of a collared artery from an animal treated with indomethacin (IndoH), **d** a section of a collared artery from a Cu(II)-Indo-treated animal, **e** a section of a collared artery from an animal treated with Cu(II)-ACM and **f** a section of a collared artery from an animal treated with Zn(II)-ACM. In **g** the results are expressed graphically as mean ± SEM (**p < 0.01)

Insertion of a carotid collar markedly up-regulated endothelial ICAM-1 expression (Fig. 2a, b, *p* < 0.01). Treatment with IndoH (3 mg/kg) led to a 79 % decrease in endothelial ICAM-1 expression [19.3 ± 1.0 vs. 4.1 ± 1.4 image units (*p* < 0.01) Fig. 2c]. Treatment with Cu(II)Indo (3 mg/kg) led to a 28 % (19.3 ± 1.0 vs. 13.9 ± 6.3 image units, *p* = 0.43) decrease in ICAM-1 expression (Fig. 2d). There was a 43 % (19.3 ± 1.0 vs. 11.0 ± 3.6 image units, *p* = 0.07) decrease in ICAM-1 expression with Cu(II)ACM (3 mg/kg), which was not statistically significant (Fig. 2e), whereas treatment with Zn(II)ACM (3 mg/kg) led to a 84 % (19.3 ± 1.0 vs. 3.1 ± 1.2 image units) (*p* < 0.01) decrease in ICAM-1 expression (Fig. 2f).

### Toxicity data

The active ingredients were well tolerated by all animals. Both macroscopic and microscopic immunohistochemical analyses revealed no evidence of gastric, hepatic or renal toxicity. Examination of the entire length of the stomach, small and large bowel showed no evidence of ulceration, gastrointestinal bleeding or inflammation. On histology there was no evidence of acute hepatitis or nephritis in any of the animals.

In this work, we have demonstrated for the first time that Cu and Zn bound anti-inflammatory agents can also be effective treatments for acute vascular inflammation in vivo. The degree of anti-inflammatory activity observed was found to be similar to that achieved with IndoH at equivalent to the FDA recommended maximum therapeutic dose (RMTD). Furthermore, this new class of anti-inflammatory agents showed no signs of organ (stomach, small and large intestine, liver, and kidney) acute toxicity at this dose.

Specifically, we observed that Cu(II)-bound Indo and ACM achieved a comparable level of inhibition of endothelial VCAM-1 expression as observed after treatment with IndoH. Interestingly, Zn(II)-bound ACM was able to decrease expression of endothelial ICAM-1 substantially, despite a lack of significant inhibition of VCAM-1 expression, which indicated that Zn(II)ACM inhibits inflammation independent of the canonical nuclear factor-κB (NF-κB) pathway. Importantly, the results of this study are comparable to a similar study in which the anti-inflammatory effects of the atheroprotective agent lipid-free apolipoprotein A-I (apoA-I) were investigated [10]. However the degree of inhibition of VCAM and ICAM achieved warrants larger studies including mechanistic studies at the MRNA level to further delineate these effects in vivo.

Treatments with both NSAIDs and coxibs are associated with significant rates of GI and cardiovascular (CV) pathology [2–4]. It was initially postulated that selective coxibs may specifically decrease GI adverse side effects. However, it is now apparent that, despite a reduction in GI complications with selective coxibs, serious CV events may occur [11]. Therefore, there is pressing clinical need for the development of effective anti-inflammatory agents with a wider safety and therapeutic window.

An early strategy to circumvent the inherent toxicity of NSAID use was by means of NSAID complexation with metals, particularly Cu. This arose from the research of Hangarter in the mid-twentieth century [12], and the hypothesis of Sorenson in the 1990s that the in vivo activity of NSAIDs might be due to binding with endogenous Cu [13], and the gastric-sparing effect of the Cu(II)-NSAID complexes. Many other Cu(II)-NSAID complexes have since been synthesised [7, 13] as well as those of other metals, such as Zn(II) [14], for the assessments of their anti-inflammatory, anti-cancer and anti-diabetic effects.

Acemetacin (systematic name: 2-[2-(1-(4-chlorobenzoyl)-5-methoxy-2-methyl-1*H*-indol-3-yl)acetoxy]acetic acid) is an IndoH prodrug with inherent anti-inflammatory activity. ACMH has been on the market for over 35 years and although its GI-protective advantage relative to IndoH is well-established [8], relatively little is known about its mode of action [15]. ACMH is known to be rapidly absorbed and converted to IndoH in vivo, with complete biotransformation within 1 h of ingestion. It has been suggested that the reduced GI toxicity of ACMH results from its lack of stimulation of leucocyte-endothelial adherence within mesenteric venules, a crucial event in the genesis of NSAID gastropathy [15], hence it is considered a suitable candidate for evaluation of its anti-inflammatory activity and non-ulcerogenic potential in a variety of conditions and diseases. Further, expansion of the current protocol to include ACMH and/or other market available NSAIDs would also be of value.

Preliminary investigations of the Cu(II) complex of ACM provide useful pointers to the relative safety and efficacy and dose–response of the complex compared with the parent NSAID. A comparable level of inhibition of cell adhesion molecule expression by IndoH and the metal complexes of Indo and ACM is indicative of the anti-inflammatory potency of all of these classes of drugs. Both VCAM-1 and ICAM-1 are important early markers of atherogenesis, and serum levels of soluble VCAM-1 and soluble ICAM-1 independently predict CV events [16]. It is likely that the anti-inflammatory effects of these compounds are, at least in part, explained by inhibition of NF-κB, where variable magnitudes of inhibition of VCAM-1 and ICAM-1 can be attributed to the differing effects on the regulators of their expression, especially in relation to the ACM compounds.

For clinical applications, it is necessary to consider the safety of profiles of these drugs, which are associated with their mode(s) of action. Cu(II)Indo exhibits an enzymatic anti-oxidant-like superoxide dismutase (SOD) activity, with an *IC*_50_ value (0.04 µM) [17], which is comparable to other potent SOD-mimetic Cu complexes [18]. Indo complexes with either Cu(II) or Zn(II) showed an improvement in GI safety compared to the parent drug in rats and horses [17], but this was more evident for the Cu(II) complex and its SOD-like activity may contribute to the reduced GI toxicity. The clinically protective effects of the Cu complexes are further supported by the lack of GI, hepatic and renal toxicities observed in the NZW rabbit model used in the current study. Moreover, in Phase 1 human clinical trials for the treatment of proctitis, no toxicity was observed with Cu(II)Indo where normally the GI toxicity of NSAIDs would preclude treatment for this condition [19].

A limitation of this study was the small numbers of animals per treatment group. This maybe of relevance to the differential inhibitory effects previously discussed. However, the degree of inhibition of VCAM-1 and ICAM-1 achieved warrants larger studies to further delineate these effects in vivo; in particular investigation of effects on vascular inflammation of a combined therapy using both Cu(II)-NSAID and Zn(II)-NSAID compared with the parent NSAID alone. Also as the model used in our experiments was one of acute vascular inflammation, further experiments investigating chronic vascular insults leading to atherosclerotic changes in the vessel wall are required. Finally, in regards to toxicity data, it is unlikely that major adverse effects would be observed after a single dose of an NSAID in a small number of animals, as used in our study. In future studies we plan to expand the dose range and duration, as well as measure liver concentrations of copper and plasma concentrations of ceruloplasmin.

In conclusion, we have demonstrated for the first time that a single oral dose of Cu(II) and Zn(II)-bound Indo-like compounds have marked anti-inflammatory effects in an acute model of vascular inflammation in vivo. This study establishes the “proof-of-concept” that such a class of drug can be effective with the additional benefit of no apparent GI, hepatic and renal toxicity. The ability of these compounds to inhibit the expression of adhesion molecules provides the initial evidence to further develop these drugs for human conditions of acute and chronic vascular inflammation and investigate their application in the treatment of other inflammation based disease states.

## Methods

### Synthesis and formulation of test compounds

**Fig. 3:**
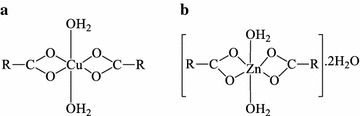
Structure of: **a** acemetacin; **b** Cu(II)ACM; and Zn(II)

Acemetacin, IndoH and zinc acetate dihydrate were obtained from Sigma Chemical Co. (St. Louis, MO, USA). Copper(II) acetate monohydrate (99.0 %) was obtained from Ajax Chemicals. All the chemicals were used without further purification for synthesis of the following active ingredients for testing: Cu(II)Indo, Cu(II)ACM, (Fig. 3a) and Zn(II) ACM [9] (Fig. 3b). Tetraglycol and sorbitan monooleate (Span 80^®^) were obtained from Sigma Chemical Co. (St. Louis, MO, USA). Fumed silica (Aerosil^®^) and medium chain triglyceride (MCT) oil were supplied by NatureVet Pty. Ltd. The MCT organogel was prepared by dissolving the active ingredient in tetraglycol (40 % w/w), Span 80 (20 % w/w) and MCT oil (adjusted to 100 % w/w), and heating the mixture to 55–60 °C. Aerosil was added to the still warm solution, and mixed vigorously to obtain a homogenous and transparent organogel. The organogel was allowed to de-gas overnight prior to administration.

### Ethics statement

Approval of the Sydney South West Area Health Service Animal Welfare Committee was obtained. Male New Zealand White (NZW) rabbits (from the IMVS, Adelaide, South Australia) weighing approximately 2.5 kg were maintained on a normal chow diet throughout the studies. A non-occlusive, silastic, peri-arterial collar was positioned around the left common carotid artery [10]. The collars were left in place for 24 h at which time the animals were sacrificed.

The formulation was administered via laparotomy under the same general anaesthesia immediately prior to collar insertion. The rabbits (*n* = 4 in each set of conditions) received either saline (positive control), IndoH (3 mg/kg), Cu(II)Indo (3 mg/kg), Cu(II)ACM (3 mg/kg), or Zn(II)ACM (3 mg/kg). The non-collared right carotid artery was used as a control for each collared artery. The dosing for these experiments was determined on the basis of our previously published animal experiments, where there was minimal organ toxicity reported [9].

The collared segment of the left carotid artery and a 30 mm segment of the contra-lateral carotid artery were collected. For immunohistochemical analysis, ring sections were cut from the arteries, fixed in cold ethanol and wax-embedded, as described previously [10].

A total of 8–10 contiguous sections (5 μm) and 20 random fields/section were analysed for each vessel. Mouse anti-rabbit vascular cell adhesion molecule (VCAM)-1 and intercellular adhesion molecule (ICAM)-1 antibodies (donated by Dr M. Cybulsky, University of Toronto) were used to assess endothelial expression of VCAM-1 and ICAM-1 as described previously [10].

Endothelial expression of VCAM-1 and ICAM-1 was determined by quantitative immunohistochemistry using ImagePro Plus 4.5 software (Media Cybernetics, Silver Spring, MD) [20]. The results, which represent the average positive staining above the threshold, set by a blinded experienced pathologist, for individual arterial sections are expressed as image units. Systematic sampling of each artery quadrant generated a single mean image unit value for staining for each artery. The mean of these values represents the amount of staining per treatment group, and was used for subsequent statistical comparison.

A Roche diagnostics kit (Basel, Switzerland) was used to determine plasma total cholesterol levels and HDL cholesterol levels as described [21]. The concentration of rabbit apoA-I was determined immunoturbidometrically [22]. These parameters were measured immediately prior to carotid surgery and at the time of sacrifice.

All results are expressed as mean ± SEM. Data obtained from digital analysis were regarded as continuous variables and satisfied the Kolmogorov–Smirnov test to determine normal distribution. Statistical comparisons were then made by unpaired two-tailed Student’s *t* tests using Graph Pad Prism Version 4.0 (San Diego, CA). A value of *p* < 0.05 was considered statistically significant.

## Abbreviations

NSAIDs: non-steroidal anti-inflammatory drugs
VCAM-1: vascular cell adhesion molecule-1
ICAM-1: intercellular adhesion molecule
COX-2: cyclooxygenase-2
ACMH: acemetacin
IndoH: a glycolic ester of indomethacin
Cu: copper
Zn: zinc
SOD: superoxide dismutase
